# Imbalance of excitation and inhibition at threshold level in the auditory cortex

**DOI:** 10.3389/fncir.2015.00011

**Published:** 2015-03-18

**Authors:** Yan Zhao, Zizhen Zhang, Xiuping Liu, Colin Xiong, Zhongju Xiao, Jun Yan

**Affiliations:** ^1^Department of Physiology, School of Basic Medical Science, Southern Medical UniversityGuangzhou, China; ^2^Department of Physiology and Pharmacology, Cumming School of Medicine, Hotchkiss Brain Institute, University of CalgaryCalgary, AB, Canada

**Keywords:** auditory cortex, *in vivo* whole cell patch, excitatory-inhibitory imbalance, thalamocortical model, minimal threshold

## Abstract

The interplay of cortical excitation and inhibition is a fundamental feature of cortical information processing. Excitation and inhibition in single cortical neurons are balanced in their response to optimal sensory stimulation due to thalamocortical feedforward microcircuitry. It is unclear whether the balance between cortical excitation and inhibition is maintained at the threshold stimulus level. Using *in vivo* whole-cell patch-clamp recording of thalamocortical recipient neurons in the primary auditory cortex of mice, we examined the tone-evoked excitatory and inhibitory postsynaptic currents at threshold levels. Similar to previous reports, tone induced excitatory postsynaptic currents when the membrane potentials were held at 70 mV and inhibitory postsynaptic currents when the membrane potentials were held at 0 mV on single cortical neurons. This coupled excitation and inhibition is not demonstrated when threshold-level tone stimuli are presented. In most cases, tone induced only excitatory postsynaptic current. The best frequencies of excitatory and inhibitory responses were often different and thresholds of inhibitory responses were mostly higher than those of excitatory responses. Our data suggest that the excitatory and inhibitory inputs to single cortical neurons are imbalanced at the threshold level. This imbalance may result from the inherent dynamics of thalamocortical feedforward microcircuitry.

## Introduction

Neurons in layers III-IV of the auditory cortex assemble auditory information from thalamocortical inputs (McMullen and de Venecia, 1993; Winer et al., 2005; Lee, 2013). As with other excitatory neural circuitry, thalamocortical excitation is coupled with inhibition, both of which are essential for cortical function involving neural computation and plasticity (Froemke and Jones, 2011; Wu et al., 2011; Chadderton et al., 2014).

Studies of visual, auditory and somatosensory cortices have demonstrated that excitation and inhibition are often coupled in single cortical neurons (Wehr and Zador, 2003; Zhang et al., 2003; Tan et al., 2004; Zhu et al., 2004; Monier et al., 2008). The degree of coupling describes the balance between excitation and inhibition in cortical information processing. In the auditory cortex, the neuronal receptive field constructed on excitatory postsynaptic conductance (EPSC) is largely mirrored by the neuronal receptive field constructed on inhibitory postsynaptic conductance (IPSC; Wehr and Zador, 2003; Wu et al., 2008; Sun et al., 2010; Kong et al., 2014). Studies in the visual cortex recently showed that the ratio of inhibition and excitation is mostly consistent across individual neurons at the thalamocortical recipient layer (Tao et al., 2014; Xue et al., 2014). These findings suggest that the excitatory and inhibitory feedforward microcircuitry is a fundamental unit of the thalamocortical system (Miller et al., 2001; Suder et al., 2002; Metherate et al., 2005; Liu et al., 2011). The inhibition in this feedforward circuitry shapes the output, i.e., firing and receptive field of the recipient neurons in layers III/IV of the auditory cortex (Wehr and Zador, 2003; Wu et al., 2008).

Of note, previous studies that examined the balance of cortical excitation and inhibition have focused on neuronal responses to optimal stimulation. The dynamics of this feedforward inhibition appears to occur in a linear manner; the degree of inhibition is largely correlated to the increase or decrease in excitation following the changes in stimulation (Wehr and Zador, 2003; Tan et al., 2004). However, the ratio of inhibition and excitation can largely decrease in response to higher sound levels in non-monotonic neurons. This suggests a level-dependent dynamics of thalamocortical feedforward excitation and inhibition (Tan et al., 2007; Wu et al., 2011). It remains unclear how cortical excitation and inhibition interact at the threshold level.

The results of extracellular studies confirm that the uncertainty of neuronal firing sharply increases at the threshold level (Heil et al., 1992; Bowman et al., 1995), which is well in accordance with psychoacoustic findings of the low detectability of sound at the hearing threshold (Viemeister, 1988). Is the cortical excitation and inhibition interaction at threshold levels distinct from that at optimal stimulus level, i.e., poor balanced or completely imbalanced? Clarification of this issue also benefits our understanding of thalamocortical feedforward circuits. Here, we recorded the EPSCs and IPSCs of layers III-IV neurons in the mouse auditory cortex in response to threshold tones by using *in vivo* whole-cell patch-clamp. We show that the excitation and inhibition of cortical neurons were largely imbalanced at the threshold levels.

## Materials and Methods

The methodologies for animal preparation, acoustic stimulation, and confirmation of the location of the primary auditory cortex in the present study are identical to those described in our previous work (Luo et al., 2008; Liu et al., 2015). The materials and methods related to *in vivo* whole-cell patch-clamp recording are described in detail. The animal protocol was approved by the Animal Care Committee at the University of Calgary (Protocol AC12-203).

### Anesthesia and Surgery

Eighteen female C57 mice of 4–5 weeks in age and weighing 17–20 g were employed in our experiments. Anesthesia for the experiments consisted of a ketamine/xylazine mixture. The first dosage of 85 mg/kg ketamine and 15 mg/kg xylazine was intraperitoneally administered. The level of anesthesia was maintained by additional dosages of ketamine (17 mg/kg) and xylazine (3 mg/kg) administered approximately every 40 min throughout the physiological experiments. Under anesthesia, the mouse’s head was fixed in a custom-made head holder by rigidly clamping between the palate and nasal/frontal bones. The scalp, muscles and soft tissues of the left skull were then removed, an opening above the auditory cortex was made using a dental driller, and the dura was gently removed. The mouse was placed on a feedback-controlled heating pad to maintain its body temperature at ~37°C. All electrophysiological experiments were performed in a soundproof and echo-attenuated chamber.

### Acoustic Stimulation

To sample the responses of auditory neurons to different frequencies and amplitudes, pure tone bursts of 20 ms duration and 5 ms rising-decay time were generated using a RP2 real-time processor (TDT, Tucker-Davis Tech., Inc., Alachua, FL, USA). The RP2 output was fed to a TDT PA5 digital attenuator. The tone frequency and amplitude were altered either manually or automatically through the TDT BrainWare software that controlled the RP2 and PA5. Tone bursts were played through a free field loudspeaker that was positioned 15 cm away from and 45° right of the mouse right ear. The output of the loudspeaker was calibrated with a Larson–Davis condenser microphone (Model 2520) and a microphone preamplifier (Model 2200C). The calibration was done without the attenuation of RP2-generated signals and the tone intensity was expressed as dB SPL (reference sound pressure: 20 µPa). The output (frequency response curve) of the loudspeaker at the frequency range of 1–50 kHz was flattened by the adjustment of RP2 output voltages (or the input voltage to the loudspeaker). Tone bursts were delivered to the mouse at a rate of 1 Hz for testing trials and at a rate of 2 Hz for data sampling. The best frequency (BF) and minimum threshold (MT) of single neurons were quickly determined by manual alteration of tone frequency and amplitude. A frequency amplitude scan (FA-scan) was then used to sample the frequency-threshold tunings of single neurons. An FA-scan consisted of 11 frequency steps and 7 amplitude steps. The intervals between steps were 1 kHz for frequency and 5 dB for amplitude. The central frequency of the scanning range was the BF and the amplitude was from 5 dB below to 30 dB above the MT. Neuronal responses to 5 identical FA-scans were used to construct the neuronal receptive fields.

### Recording in the Primary Auditory Cortex (AI)

Glass pipette electrodes of ~1 µm in diameter at the tip and 7–9 MΩ in tip impedance were used for voltage-clamp recordings. The electrode was connected with the Multiclamp 700B amplifier (Molecular Device, Sunnyvale, USA) via a headstage (including an electrode holder). To block action potential firing and improve space clamp, the electrode pipettes were filled with a solution containing sodium channel blocker QX-314 and Cesium. The solution (in mM) consisted of 125 Cs-gluconate, 5 TEA-Cl, 4 MgATP, 0.3 GTP, 10 phosphocreatine, 10 HEPES, 0.5 EGTA, 3.5 QX-314 (sodium channel blocker), and 2 CsCl. The pH was adjusted to 7.2 using cesium hydroxide or Gluconic acid and the osmotic pressure was approximately 290 mOsm.

Once the location of the AI was confirmed by recording tone-evoked responses at 5–8 loci of the exposed cortex, the glass pipette electrode was perpendicularly inserted about 400 µm below the surface of the cortex. During electrode penetration, a positive pressure between 100–200 mbar was applied to the electrode to avoid the contamination of the pipette tip. The electrode was advanced by 0.8 µm per step to within 400–700 µm below the cortical surface. A positive square pulse (10 mV and 100 ms) was delivered via the electrode and monitored on Clampex 10.4 data acquisition software (Molecular Device, Sunnyvale, USA) for measuring the changes in tip impedance. The positive intra-pipette pressure was released when the tip impedance sharply increased by ~20%. A successful cell attach was indicated by a giga-ohm seal following pressure release or a slight negative pressure was applied to the pipette.

Upon the successful sealing, additional negative pressure was applied to break the cell membrane and to achieve whole-cell patch configuration on a single neuron. The whole cell capacitance and the initial series resistance (21–50 MΩ) were compensated to achieve a series resistance of 16–40 MΩ. This study used the voltage-clamp mode. The holding membrane potential was set at 70 mV for recording excitatory postsynaptic currents (EPSC) and at 0 mV for recording inhibitory postsynaptic currents (IPSC). Three experimental protocols followed and are described below. The bioelectrical signals were fed to the DigiData1550 (Molecular Device, Sunnyvale, USA) and RP2 (TDT, Tucker-Davis Tech., Inc., Alachua, USA) via the Multiclamp 700B amplifier. The signals to the DigiData1550 were filtered by a 4-kHz lowpass filter and those to the RP2 were filtered by a 2–30 Hz bandpass filter. Data were simultaneously sampled by Clampex (Molecular) and BrainWare (TDT) software at a sampling rate of 10 kHz. The clampex saved the EPSC/IPSC waves with the original current values and the BrainWare saved the waves with tone information.

Three data groups were sampled. The first measured the input resistance and the voltage-dependent postsynaptic currents. The input resistance was the holding membrane potential (−70 mV) divided by the measured current (mV/pA). The tone-evoked postsynaptic currents were sampled at holding potentials of −90, −70, −30 and 0 mV. The current values were measured using two time windows: 0–1 ms and 5–7 ms from the onset of the response at −90 mV holding potential. The second data group recorded EPSCs responses to tones at various frequencies and amplitudes (FA-scan) under a −70 mV holding potential. The third data group recorded IPSCs in responses to an FA-scan under a 0 mV holding potential. Data were excluded if the recordings were incomplete in any of the three data groups.

### Data Processing and Statistical Analysis

The data analyses were based on the synaptic conductance derived from the recorded synaptic current using the formula

(1) I(t, V)=ge(t)(V−Ee)+gi(t)(V−Ei)

The *ge(t)* and *gi(t)* were the time *t* function of excitatory and inhibitory synaptic conductance (Tan et al., 2004). In the formula, the *V* and *I* are the membrane potential and current at different times. In our methodology, *Ee* = 0 mV and *Ei* = −70 mV (Borg-Graham et al., 1998; Hirsch et al., 1998; Anderson et al., 2000), which were dependent on the ionic concentration in the intracellular solution and cerebrospinal fluid. The unit of conductance is nano Siemens (nS). We used two criteria to determine a tone-evoked EPSC or IPSC of a single neuron. The first was that the absolute peak value of a negative-going (EPSC) or positive-going (IPSC) waveform was at least 15% larger than the averaged fluctuation of the baseline. The second was that the EPSC or IPSC peak fell within the time-window of the largest EPSC or IPSC waveform induced by a tone with identical frequency but optimal amplitude. Based on the tone-evoked EPSCs and IPSCs, the neuronal minimal threshold (MT) was the lowest tone level that could induce tone-evoked EPSC and IPSC. The neuronal BF was the tone frequency to which the neuron showed EPSC or IPSC at the MT level. If a neuron showed responses to more than one frequency at the MT level, the BF was the one that induced the largest EPSC or IPSC.

The EPSC and IPSC waveforms were characterized using 5 parameters including latency, peak value, peak time, rising-slope and 50% duration. The latency was the crossing point of the baseline and rising-slope line of the waveform. The peak value and time were the differences between the amplitudes and times measured at the largest point and those at the beginning point (latency) of the EPSC or IPSC waveform. The rising-slope was the peak value divided by the peak time. The 50% duration was the width of the EPSC or IPSC waveform measured at the 50% peak value.

Data were expressed as a mean ± standard deviation. A paired *t*-test was used to compare the different data groups. A *p*-value less than 0.05 was considered statistically significant.

## Results

Complete sets of data were successfully sampled in 18 AI neurons. Since the recording area was strictly limited to a range of 400 µm to 700 µm below the brain surface, these neurons were considered to be within the thalamocortical recipient layer of the AI.

**Figure 1:**
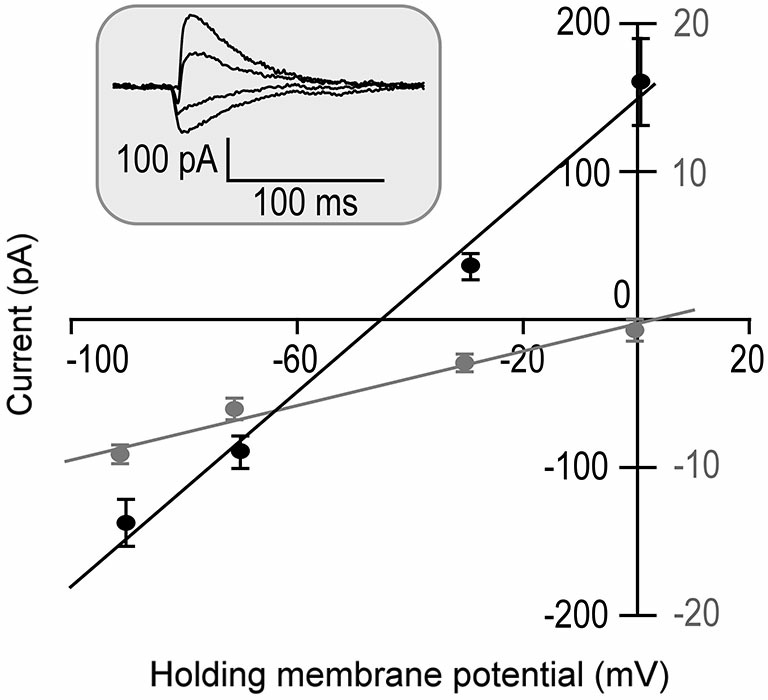
**Tone-evoked postsynaptic currents (Inset) are plotted as the function of holding membrane potentials in one neuron (repeated 5 times)**. Since the IPSC commonly has a 1–4 ms delay from the EPSC (Wehr and Zador, 2003), two time windows of 0–1 ms (Gray symbols) and 5–7 ms (Black symbols) from the response onset were selected for EPSC and IPSC measurements. The postsynaptic currents were linearly correlated to the holding potentials (*R^2^* = 0.98 and *R^2^* = 0.99, respectively).

The input resistance was first measured following a successful whole cell patch. Fifteen out of 18 recorded neurons showed relatively lower input resistances when the membrane potentials were held at −70 mV. On average, it was 205.49 ± 123.42 MΩ. The other three neurons showed larger input resistances. On average, the input resistance of these three neurons was 744.73 ± 620.60 MΩ at −70 mV. They were statistically different (*p* < 0.001). The direction of tone-evoked postsynaptic currents changed from negative to positive following the increase in the holding potential from −90 mV to 0 mV. An example shown in Figure 1 demonstrates that the neuron was well clamped. A tone (at neuronal BF) of 70 dB SPL induced excitatory postsynaptic currents (EPSCs) when the holding potentials were at −90 and −70 mV. The tone induced a small EPSC followed by large inhibitory postsynaptic currents (IPSCs) when the holding potential was at −30 mV and induced a pure IPSC when the holding potential was at 0 mV.

**Figure 2:**
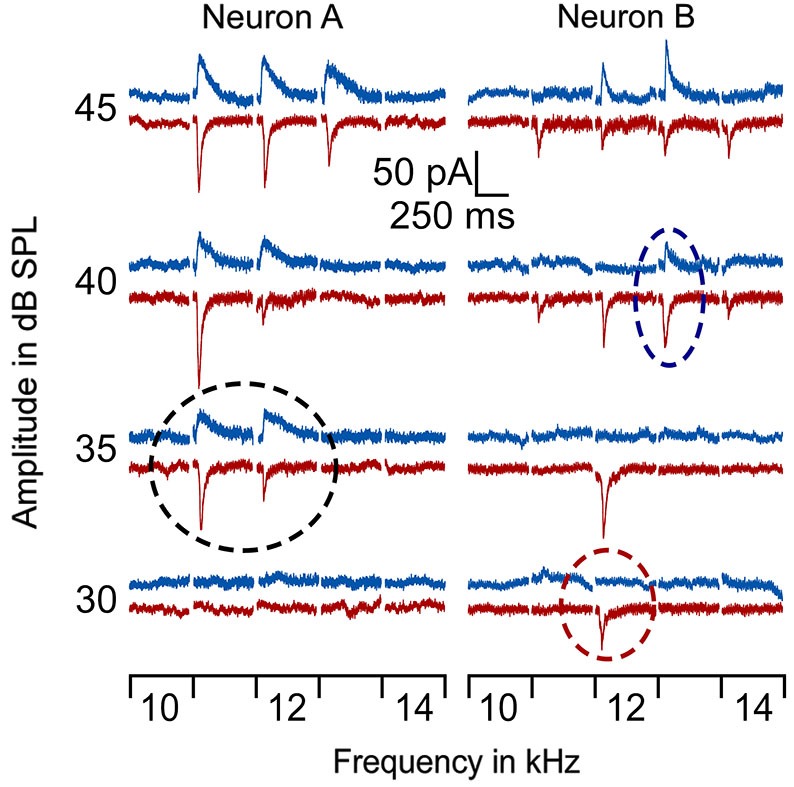
**Examples of EPSCs and IPSCs of 2 AI neurons in response to tones around BF/MT**. The BF/MT of EPSC and IPSC were identical in Neuron A (left) but not in Neuron B (right) as indicated by the dashed circles. Red lines represent EPSC and blue lines represent IPSC. The black circle shows the balanced EPSC and IPSC at MT (35 dB SPL) for neuron A. The red circle shows the MT of EPSC at 30 dB SPL and the blue circle, the MT of IPSC at 40 dB SPL.

**Figure 3:**
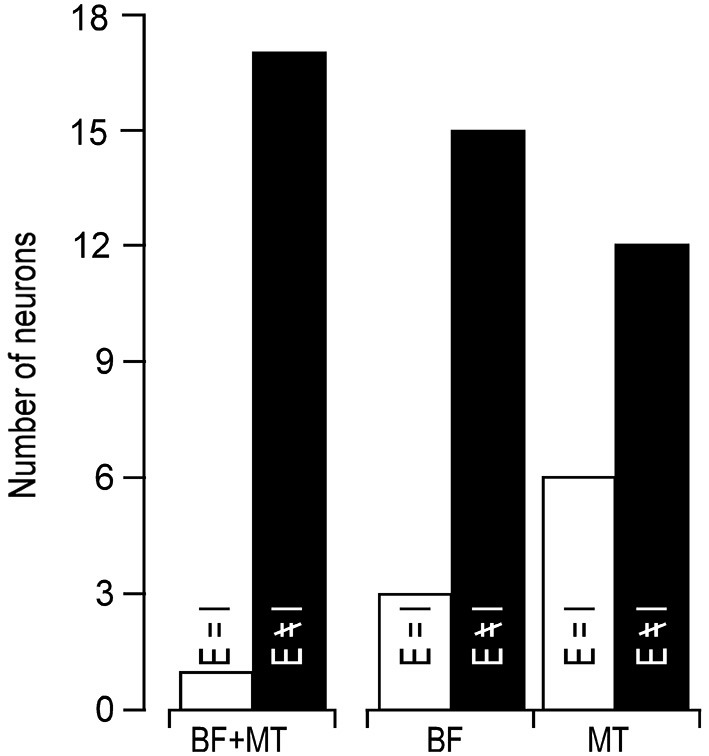
**Number of neurons showing identical (open bars) and different (filled bars) BF/MT (left), BF alone (middle), MT alone (right) between EPSC and IPSC**.

**Figure 4:**
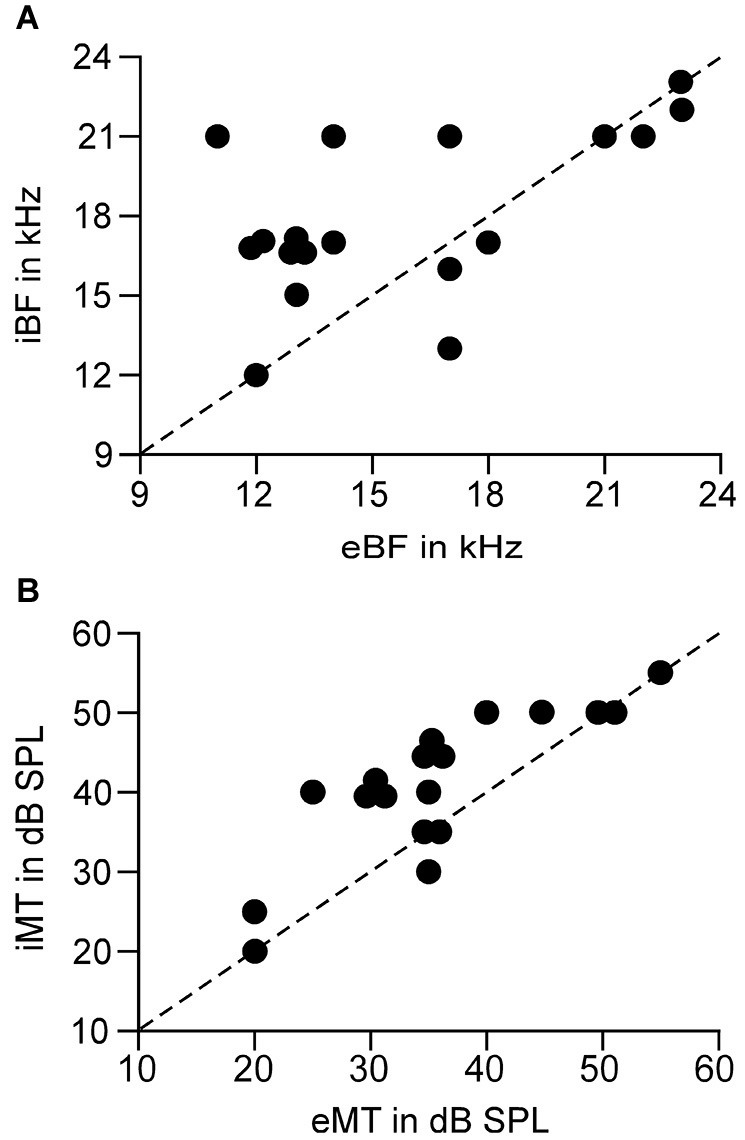
**Correlation of the BFs (A) and MTs (B) between the EPSC and IPSC of a single neuron**. Dashed line is the diagonal. Circles on the diagonals show the identical BF or MT between EPSC and IPSC. eBF/iBF: BF of EPSC and IPSC. eMT/iMT: MT of EPSC and IPSC.

In line with previous reports (Tan et al., 2004; Wu et al., 2008), tone induced EPSC when the membrane potential was held at −70 mV and induced IPSC when held at 0 mV. The tone-evoked EPSCs and IPSCs were coupled in single AI neurons at most frequencies and amplitudes within the neuronal receptive field. The EPSC/IPSC coupling however, was limited at the threshold level. Two examples are shown in Figure 2. Neuron A showed a MT at 35 dB SPL. At this level, clear EPSCs and IPSCs were induced by 11 kHz and 12 kHz tone stimuli. Since the EPSC and IPSC to 11 kHz were larger than those to 12 kHz, the BFs and MTs of tone-evoked EPSC and IPSC were respectively 11 kHz and 35 dB SPL, i.e., identical in the BFs and MTs between EPSC and IPSC. In other words, this neuron had a balanced EPSC and IPSC at the threshold level. In contrast, the BF and MT of EPSC in Neuron B were different from those of IPSC (12 kHz and 30 dB SPL vs. 13 kHz and 40 dB PSL). The tone induced EPSC but did not induce IPSC at 12 kHz and 30 dB SPL, illustrating an imbalance of EPSC and IPSC at the threshold level. Out of 18 sampled AI neurons, only 1 (5%) neuron showed balanced EPSC and IPSC while 17 (95%) neurons showed imbalanced EPSC and IPSC (Figure 3, left). The number of imbalanced neurons was much greater than that of balanced neurons. The imbalance between EPSC and IPSC of single AI neurons appeared mostly related to the difference in frequency tunings. Out of these neurons, only 3 AI neurons had EPSC BF (eBF) equal to IPSC BF (iBF) and 15 AI neurons had different eBF and iBF. The number of AI neurons with identical EPSC MT (eMT) and IPSC MT (iMT) was also lower than that with different eMT and iMT (6 vs. 12, Figure 4B).

The analysis of the relation between eBFs and iBFs indicated that the AI neurons had iBF higher than eBF in most cases. Out of 15 AI neurons in which eBFs were different from iBFs, the iBF was higher than eBF in 10 neurons and the iBF was lower than eBF in 5 neurons. Notably, 10 AI neurons had eBF and iBF difference by 3 kHz or higher and 3 neurons showed the difference by 5 kHz or higher (Figure 4A). On average, the difference between eBF and iBF was 3.11 ± 2.67 kHz, *p* < 0.001, ranging from 0 kHz to 7 kHz, (*n* = 18). Taking into account the hearing range of the C57 mouse (Yan and Zhang, 2005), this difference was significantly large.

Similar to the difference of eBF and iBF, 12 AI neurons had different eMT and iMT. Eleven of them showed higher iMT than eMT and only 1 neuron had lower iMT than eMT. It is also notable that the iMT was 10 dB or higher than eMT in 8 neurons (Figure 4B). On average, the difference between eMT and iMT was 5.83 ± 4.92 dB, *p* < 0.001, ranging from 0 to 15 dB (*n* = 18).

The EPSC and IPSC waveforms at the BFs and MTs were characterized using five parameters including latency, peak value, peak time, rising-slope and 50% duration based on postsynaptic conductance converted from the postsynaptic current. For simplicity, the EPSC and IPSC also represent the excitatory and IPSC. The IPSC latency was significantly longer than EPSC latency (45.67 ± 20.82 ms vs. 31.26 ± 11.90 ms, *p* < 0.05). The rising-slopes of IPSC and EPSC were respectively 0.95 ± 0.59 nS/ms and 0.40 ± 0.34 nS/ms, which were not statistically different (*p* > 0.05). The two components of rising-slope were peak value and time. The peak value of IPSC was also significantly larger than that of EPSC (20.48 ± 11.24 nS vs. 10.25 ± 6.53 nS, *p* < 0.05) but the difference in peak times was statistically insignificant (25.22 ± 13.26 ms vs. 31.20 ± 14.73 ms, *p* > 0.05). Of interest, the 50% duration of IPSC was significantly longer than that of EPSC (60.32 ± 22.43 ms vs. 47.69 ± 6.43 ms). It is also noteworthy that the IPSC had longer latency, higher peak and longer duration than the EPSC but its rising-slope was similar to the EPSC.

## Discussion

Our data clearly demonstrated that the coupling of cortical excitation and inhibition varied from neuron to neuron at the level of threshold stimulation. Only 5% neurons showed balanced excitation and inhibition while 95% neurons showed imbalanced excitation and inhibition (Figures 2, 3). Furthermore, 83.33% neurons showed different BFs of EPSC from those of IPSC (Figure 4A) and 61.11% neurons showed higher thresholds of inhibitory responses than those of excitatory ones (iMT > eMT, Figure 4B). These findings suggest that the excitation and inhibition are imbalanced when the stimulus only reaches the threshold levels of single cortical neurons. In contrast, the excitation and inhibition were well balanced at the BF of 30 dB above the MT. Our data could not directly differentiate whether the sampled neurons in this study were excitatory or inhibitory neurons. Since only 15–25% of neurons in the auditory cortex are inhibitory (Hendry et al., 1987; Prieto et al., 1994), it is likely that the majority of our samples were from excitatory neurons. This is also supported indirectly by our data of input resistances; 15 out of 18 recorded neurons demonstrated a significantly lower input resistance than the other three.

At a stimulus level sufficiently higher than the threshold, our data and many studies with *in vivo* whole-cell patch-clamp recording demonstrate that a tone-evoked EPSC is always balanced with a tone-evoked IPSC (Wehr and Zador, 2003; Tan et al., 2004). The balance consolidates a long-standing notion of thalamocortical feedforward excitation and inhibition; this feedforward microcircuitry contributes to the integration of thalamocortical information and shapes the output of target neurons (Wehr and Zador, 2003; Wu et al., 2008). Our data together with other findings suggest that the function of this microcircuitry at various sound levels should be dynamic instead of static or mechanical. As sound levels increase, the excitation and inhibition undergo imbalance (Figure 2) to balance (Wehr and Zador, 2003; Tan et al., 2004) and even to unbalance again if the single neuron has a non-monotonic level-rate function (Wu et al., 2006; Tan et al., 2007). A challenging but inevitable issue here is how to explain the threshold-level imbalance on the basis of thalamocortical feedforward microcircuitry. Specifically, two questions must be answered; one is why is the IMT higher than, equal to or lower than EPSC and the other is what causes the BFs differences.

An important distinction emerges from the observations of cortical excitatory and inhibitory neurons in response to a tone or thalamic stimulus in either *in vivo* or *in vitro* preparations. Cortical inhibitory neurons show more robust responses, i.e., larger postsynaptic potential and higher firing rate, to thalamocortical inputs than excitatory neurons (Bowman et al., 1995; Cruikshank et al., 2007; Schiff and Reyes, 2012). The synaptic transmission from GABAergic neuron to excitatory neuron is very efficient, and even one action potential is enough to induce changes in postsynaptic potential (Hull et al., 2009; Bagnall et al., 2011). In line with these evidences, our data showed that the IPSC had longer latency and higher peak than but similar slope to the EPSC. The implication here is that greater responsiveness of inhibitory neurons to thalamocortical inputs means that cortical excitatory neurons may have an IMT equal to or even lower than the EMT. This is apparently not the case. Our data and examples presented in other studies (Tan et al., 2004; Wu et al., 2008; Sun et al., 2010, 2013; Zhou et al., 2010; Li et al., 2014) showed that most cortical excitatory neurons have the MT of IPSCs higher than that of EPSCs. To solve this puzzle, one must examine the intricacies of the thalamocortical feedforward circuitry.

The fundamental circuit of thalamocortical feedforward excitation and inhibition consists of the direct projection of the thalamic neuron to a cortical excitatory neuron and the direct collateral projections to a cortical inhibitory neuron that in turn sends the inhibitory projection back to the excitatory neuron targeted by the same thalamic neurons (Swadlow, 2003; Sun et al., 2006). Three important facts should be considered. The first is that the axons of auditory thalamic neurons primarily terminate at the small or distal dendrites of non-GABAergic neurons. Only a small number of axons reach GABAergic neurons and typically synapse onto the large or proximal dendrites and cell body (Smith et al., 2012). The second is that cortical excitatory neurons receive inputs from thalamic neurons that have similar tuning properties while the inhibitory neurons receive inputs from thalamic neurons that exhibit a wider range of tuning properties (Simons and Carvell, 1989; Winer et al., 2005). This suggests that the thalamocortical projections to cortical excitatory neurons are restricted in single frequency channels while those to inhibitory neurons have the inputs from various frequency channels. The third is that the single thalamocortical synapses onto both excitatory and inhibitory neurons appears weak in function and that the synchronous activities of multiple thalamocortical inputs are required to drive the cortical neurons (Bruno and Sakmann, 2006).

Taken together, these features of the thalamocortical circuit allow us to outline an enriched model of thalamocortical feedforward excitation and inhibition (Liu et al., 2011, 2015; Wu et al., 2011). Four new properties appear plausible. Several thalamocortical excitatory pathways share a feedforward inhibitory pathway. In other words, the cortical inhibitory neurons receive thalamocortical inputs from different frequency and amplitude channels while cortical excitatory neurons receive inputs primarily from single frequency and amplitude channels. Secondly, synchronous activities of thalamocortical inputs are required to drive the postsynaptic activities of both cortical excitatory and inhibitory neurons. Thirdly, thalamocortical synapses onto inhibitory neurons exhibit relatively high efficiency. Finally, cortical inhibitory neurons project back to all cortical excitatory neurons. These excitatory neurons and inhibitory neurons share the inputs from the same thalamic neurons that belong to different frequency/amplitude channels. This enriched model could account for the differences in a number of properties between the EPSC and IPSC of single cortical neurons. The longer latency of the IPSC indicates more synaptic relays for inhibition. The similar rising-slopes of the IPSC and EPSC suggest the efficiencies of the excitatory and inhibitory synapses on the target excitatory neuron are relatively uniform. The longer IPSC duration is possibly more interesting, suggesting that the thalamocortical inputs to GABAergic neurons are relatively less synchronized than those to excitatory neurons. This weaker synchronization could result from diverse thalamocortical inputs originating from different frequency/amplitude channels as proposed in this model. Our model does not exclude the possibility of the varied strengths of the involved synapses; this may also contribute to the imbalance of cortical excitation and inhibition at the threshold level.

Balanced excitation and inhibition of single cortical neurons play a critical role in shaping temporal processing, which leads to more uniform timing of neuronal action potentials, i.e., more phasic firing (Wehr and Zador, 2003). The imbalance of cortical excitation and inhibition may underlie the larger variation in firing probability and the timing of cortical neurons in response to threshold sound (Haider et al., 2006; Higley and Contreras, 2006). Our ongoing investigations of imbalanced threshold-level excitation and inhibition significantly enhance the knowledge of sensory information processing and neural plasticity development in the auditory cortex (Xiong et al., 2011).

## Conflict of Interest Statement

The authors declare that the research was conducted in the absence of any commercial or financial relationships that could be construed as a potential conflict of interest.
